# Nursing Surge Capacity Strategies for Management of Critically Ill Adults with COVID-19

**DOI:** 10.3390/nursrep10010004

**Published:** 2020-09-08

**Authors:** Abbas Al Mutair, Anas Amr, Zainab Ambani, Khulud Al Salman, Deborah Schwebius

**Affiliations:** 1 Research Center, Dr. Sulaiman Al Habib Medical Group, Riyadh 91877, Saudi Arabia; dg.schwebius@gmail.com; 2 Nursing School, University of Wollongong, Wollongong, NSW 2522, Australia; 3 Patient Safety Center, Riyadh 9264, Saudi Arabia; aamr@spsc.gov.sa; 4 Nursing College, King Saud Ben Abdulaziz University for Health Sciences, Al-Hofuf 32641, Saudi Arabia; ambaniz@ksau-hs.edu.sa; 5 Nursing Department, Al-Jaber Hospital for Ear, Nose, Throat and Eye, Ministry of Health, Al-Hofuf 36422, Saudi Arabia; kaalsalman@moh.gov.sa

**Keywords:** COVID-19, coronavirus 2019, ICU surge capacity, nursing surge capacity and strategies

## Abstract

**Background:** There is a vital need to develop strategies to improve nursing surge capacity for caring of patients with coronavirus (COVID-19) in critical care settings. COVID-19 has spread rapidly, affecting thousands of patients and hundreds of territories. Hospitals, through anticipation and planning, can serve patients and staff by developing strategies to cope with the complications that a surge of COVID-19 places on the provision of adequate intensive care unit (ICU) nursing staff—both in numbers and in training. Aims: The aim is to provide an evidence-based starting point from which to build expanding staffing models dealing with these additional demands. **Design/Method:** In order to address and develop nursing surge capacity strategies, a five-member expert panel was formed. Multiple questions directed towards nursing surge capacity strategies were posed by the assembled expert panel. Literature review was conducted through accessing various databases including MEDLINE, CINAHL, Cochrane Central, and EMBASE. All studies were appraised by at least two reviewers independently using the Joanna Briggs Institute JBI Critical Appraisal Tools. **Results:** The expert panel has issued strategies and recommendation statements. These proposals, supported by evidence-based resources in regard to nursing staff augmentation strategies, have had prior success when implemented during the COVID-19 pandemic. **Conclusion:** The proposed guidelines are intended to provide a basis for the provision of best practice nursing care during times of diminished intensive care unit (ICU) nursing staff capacity and resources due to a surge in critically ill patients. The recommendations and strategies issued are intended to specifically support critical care nurses incorporating COVID-19 patients. As new knowledge evidence becomes available, updates can be issued and strategies, guidelines and/or policies revised. **Relevance to Clinical Practice:** Through discussion and condensing research, healthcare professionals can create a starting point from which to synergistically develop strategies to combat crises that a pandemic like COVID-19 produces.

## 1. Introduction

The recent viral outbreak initiated from Wuhan, China, has now crossed all borders and has spread into more than 224 countries [1]. The outbreak is caused by a novel strain of coronavirus which is very much similar to the SARS-CoV that resulted in the SARS outbreak [2]. Initially, this new coronavirus was named as 2019-nCoV and then was renamed as Severe Acute Respiratory Syndrome Corona Virus-2 (SARS-CoV-2) by the International Committee on Taxonomy of Viruses (ICTV) [1]. The World Health Organization (WHO) has termed COVID-19 for the disease associated with the infection caused by SARS-CoV-2 [3,4,5]. The coronavirus has been the focus of global attention after its first report in Wuhan, China, in December 2019. COVID-19 has rapidly spread all over the globe [6]. According to the WHO, as of July 13th, 2020, there were 12,880,565 confirmed cases of COVID-19 including 568,573 reported deaths globally [6]. 

SARS-CoV-2 transmission of the virus from human to human has become evident and documented in multiple published studies [7]. The mode of transmission for COVID-19 virus was initially thought to occur through droplets of saliva or discharge from the nose when an infected person coughs, sneezes, or speaks [8]. Recent studies have shown that the virus may remain suspended in the air, in the form of aerosols, for upwards of several hours [8]. However, the WHO maintains that these studies do not replicate typical cough conditions as they were produced with high-powered jet nebulizers and they have not altered their recommendations as of the date of this article submission [9]. Although maintaining at least one-meter of distancing is recommended by the WHO, it has been suggested by a recent study published in *The Lancet,* that more protection can be had if that distance is extended to two meters or more if possible. The avoidance of large gatherings, the wearing of masks, wearing of eye protection and regular hand-washing or use of alcohol-based hand rub is important to stop the transmission cycle and minimize the risk of infection [8]. Until now, there are no approved specific vaccines or treatments for COVID-19 [7]. Maintaining at least one-meter distance among individuals and regular hand-washing or using alcohol-based hand rub is important to stop the transmission cycle and minimize the risk of infection [10]. The most common symptoms of COVID-19 include fever, dry cough, and tiredness [7]. An infected person may develop some less common symptoms such as pain, sore throat, loss of taste or smell, headache, and diarrhea [7]. In critical cases, serious symptoms may appear as difficulty breathing, chest pain, and loss of speech or movement [7]. The foremost problem with COVID-19 is that a major proportion of infected persons do not exhibit or experience symptoms and hence serve as asymptomatic carriers. COVID-19 virus can transmit from symptomatic and asymptomatic carriers to other people and cause the disease [10]. 

Frontline healthcare workers in general, and nursing staff more specifically, as the backbone of any healthcare system, face additional burdens and hazards as they respond to the current COVID-19 pandemic. These burdens include exposure to pathogens, physical and psychological distress, fatigue, long working hours, and burnout [11]. The COVID-19 pandemic denotes a unique challenge to intensive care services. During a pandemic, the principal difficulties surround the preparation of intensive care units (ICU) and healthcare workers for the expected surge in caseload [11]; likely, complicated by workforce challenges including potential difficulty in maintaining standard staffing ratios [11]. In fact, frontline workers tend to get more severely ill than patients and it is not based on expectation of their ages [12]. This could be due to higher viral load exposure but also the high level of stress acting to depress the immune systems of overtaxed frontline healthcare workers [12]. Healthcare workers may experience severe symptoms and lose their ability to work due to admission or death, or they may experience mild symptoms and go under self-isolation for 14 days or more [12]. In both cases, healthcare facilities are expected to lose a considerable number from their manpower and functionality at this critical time [12]. The COVID-19 pandemic has placed a huge strain on health systems due to the increasing number of patients requiring acute and critical health care, staff, hospital beds, supplies, and resources [12]. 

To deal with this crisis, some countries developed plans and guidelines for crisis management [3]. The management is targeting the scarcity of staff, space, beds, and supplies [3]. Some of these plans were made by the national (governmental) level such as inviting all healthcare professionals to re-join the workforce, relaxing some of the licensing requirements, and accelerating credentialing processes to rapidly incorporate healthcare workers into working in hospitals [11]. Other plans were made at the hospital levels such as developing triage protocol to reallocate human and medical resources to equitably meet the needs of patients [13,14]. Triage process often starts by inventory of potential ICU resources, such as ventilatory capacity in the hospital, and then follows an algorithm for screening and admission [13]. Periodic patient assessment is necessary to check if there is any change in patients’ needs in order to transfer, admit, or discharge patients [14]. Triage protocols may also be developed at a regional level to allow for communication and resource sharing among all hospitals in one region [14]. This strategy gives more opportunities for better utilization of resources [14]. 

The goal of nursing surge capacity is to find wise ways to augment and extend the hospital workforce; to allocate healthcare resources in an ethical, rational, and organized method to do the greatest good for the greatest possible number of patients [14]. In order to combat the complications that the pandemic threatens to level of care, a decision was made to develop nursing surge capacity recommendations and strategies for management of critically ill patients with COVID-19 in the ICU. The objectives of these strategies are to provide guidance and recommendations in order to help nursing administrators and leaders to prepare for a COVID-19 pandemic in ICU. 

## 2. Methods

### 2.1. Data Sources 

**Figure 1:**
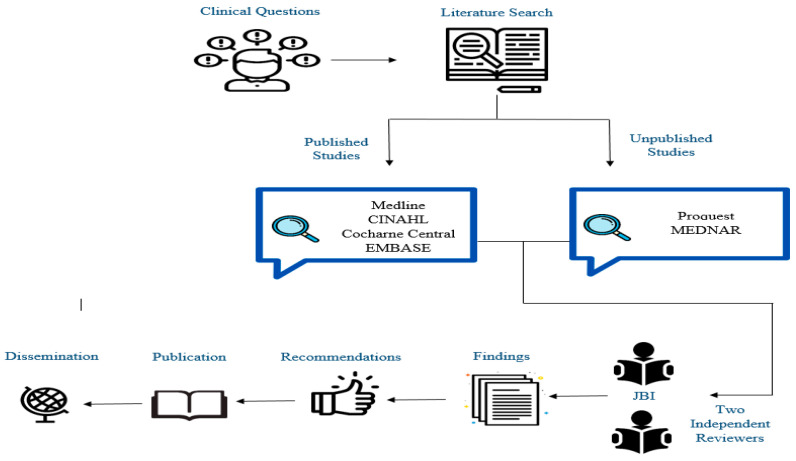
Literature searching and recommendations’ development framework.

The search strategy aimed to find published studies in MEDLINE, CINAHL, Cochrane Central, and EMBASE from December 2019 through March 2020 (Figure 1). The keywords used were: *COVID-19*, *coronavirus 2019*, *ICU surge capacity*, *nursing surge capacity*, and *strategies*. The filters applied included “humans”, “last 10 years”, and “English language”. The unpublished studies were searched in ProQuest and MEDNAR. 

### 2.2. Quality Assessment of Extracted Data

Initially, all titles and abstracts were screened independently by at least two reviewers. All full texts of the studies which passed through the initial stage were retrieved and assessed against the review inclusion criteria in detail. These eligible studies were again appraised by at least two reviewers independently using the Joanna Briggs Institute JBI Critical Appraisal Tools [15]. The JBI appraisal has different checklists to be applied against different study designs. The instrument consists of 10 items that assess the methodological quality of a study and determines the extent to which a study has addressed the possibility of bias in its design, conduct, and analysis. The results of the JBI appraisal have been taken into full account and used to inform the synthesis and interpretation of the results of the recommendations (Figure 1).

**Figure 2:**
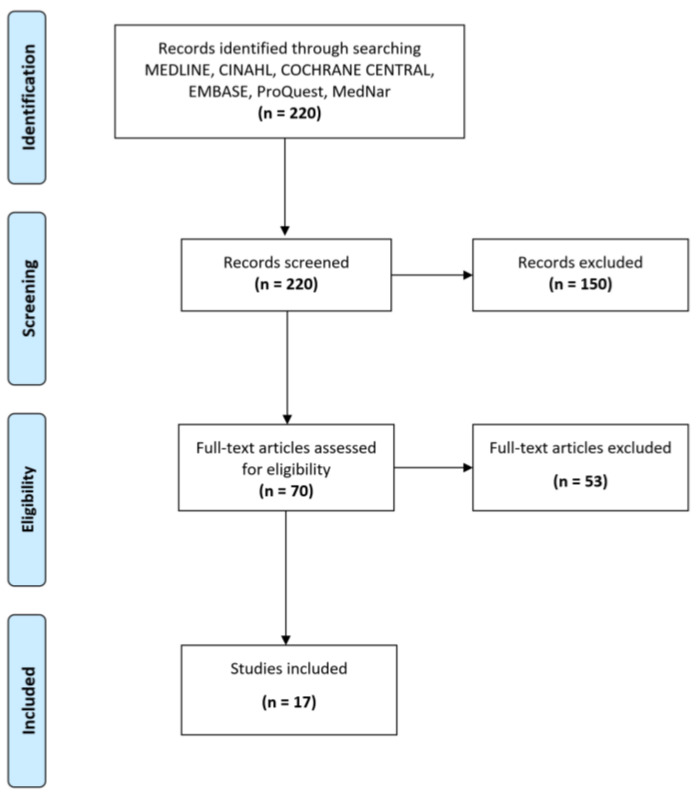
Flow diagram.

A total of 220 studies were retrieved. After reading the titles and abstracts, 150 studies were excluded. After reading the full articles, a total of 53 articles were excluded and 17 articles were included which met the inclusion criteria (Figure 2). All identified publications were collated and fed into Endnote X10 software. The evidence-based strategies issued are to support critical care nurses to manage critical patients in the intensive care unit during the COVID-19 pandemic. Four recommendations and rationales were issued by the expert panel based on evidence.

## 3. Strategies to Meet Nursing Surge Capacity during the COVID-19 Pandemic

### 3.1. Recommendation 1: Regular Patient-to-Nurse Ratio

**Figure 3:**
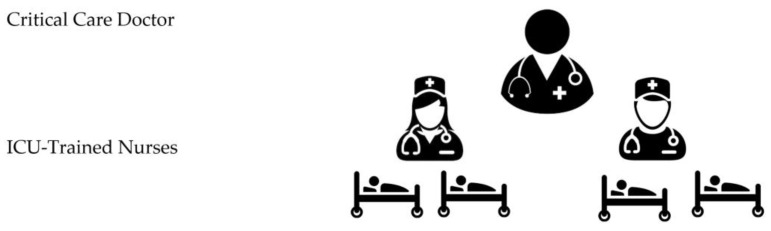
Standard ICU staffing model. (1 ICU-trained nurse: 2 patients).

When able to, recommend nursing staffing (1:1 or 1:2) in the ICU during the COVID-19 pandemic to provide high-quality patient care, improve safety, have fewer complications, and better outcomes (Figure 3). This should be followed until such time that the surge is felt. At that time, progression to Recommendation 2 will be made. 

#### Rationale

Matching patient needs with adequately trained nurses and maintaining safe patient-to-nurse ratio is essential to ensure the provision of safe and high-quality patient care. As such, nurse staffing ratios in critical care units is an important aspect when planning care [16]. The literature on nursing ratios in ICU has confirmed the relationship between ICU nurse staffing and patient outcomes. The reviewed studies confirm that a higher number of registered nursing staff to patient ratio (1:1 or 1:2) is highly associated with improved patient safety and better outcomes [13]. In the U.S. and Canada, the nurse-to-patient ratio in ICU stays close to (1:1.5) at both time points. Western Europe and Latin America had lower nurse staffing, especially at night, with an overall ratio of (~1:1.8) [17]. Note that this is the preferable situation when applicable or during non-pandemic times.

Additionally, critically ill patients require the care of nurses who have specialized knowledge and skills and who are given enough time to provide that care safely. Appropriate staffing ensures effective pairing of patient/family needs with the assigned nurse’s knowledge, skills, and abilities. In fact, evidence confirms that the likelihood of serious complications and mortality rates increase when fewer registered nurses (RNs) are assigned to care for patients [13,18,19]. Similarly, a considerable amount of research indicates healthy work environments and better patient outcomes when a higher percentage of patient care tasks are provided by RNs [20]. 

### 3.2. Recommendation 2: Finding Alternate Staff from Internal and External Resources to Support ICU Staff during Crisis Time

#### Rationale

Most countries that have already been hit hard by COVID-19, attempted to increase the supply of healthcare. Having care directed by trained and experienced ICU nurses is an effective way to provide high-quality care for critically ill patients [21]. However, during crisis times, the number of ICU nurses cannot accommodate a large number of patients. Additional personnel can be identified internally through the scale-back of elective and non-urgent services in the hospital. As elective surgeries are placed on hold, nurses from areas like the Surgical ICU, Endoscopic units, Step-down units, Post Anesthesia Care Unit (PACU), and Pre-Op become available for ICU staffing needs. These nurses should be the first choice to augment ICU staffing and expand ICU beds during pandemics such as COVID-19, as their skills are most readily transferable, thereby having the potential to increase the critical care capacity of the hospital in the safest way possible. To expand the staffing capacity further, hospitals may consider external searching resources to identify and recruit ICU nurses who had transitioned to ambulatory care settings and other nurses from community care settings to support ICU staff during the crisis [7]. Additionally, other qualified medical professionals can be recruited to safely manage the care of mechanically ventilated patients. Anesthesiologists and physicians who have ventilator management experience are potential resources to supplement ICU care teams. With minimal orientation, they can easily support respiratory therapists and nurses to achieve safe ventilatory support to those requiring it [7]. Other potential caregiver support could include students in medical, nursing, and other health education programs who are nearing the end of their studies. Many would be suitable for providing services to patients or helping to respond to public concerns through telephone hotlines [21]. 

### 3.3. Recommendation 3: Implement a Team-Based Approach (Tiered Staffing Strategy or Care Team Model) to Manage Critically Ill Patients 

**Figure 4:**
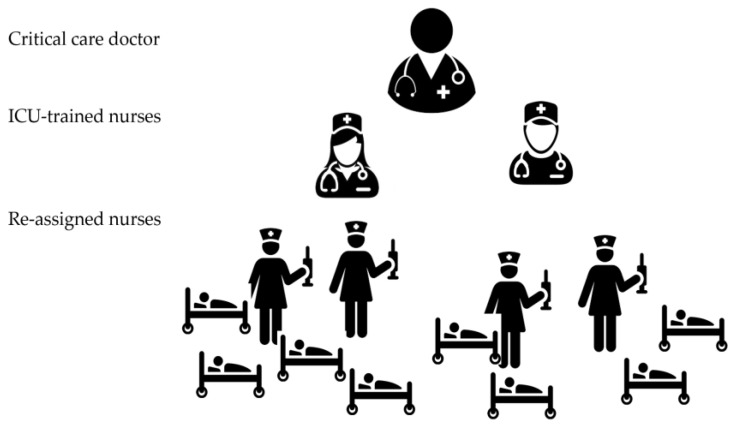
ICU tiered staffing strategy for COVID-19 pandemic; (1 ICU-trained nurse: 2 re-assigned nurses: 4 patients). This model can be expanded on a needs basis as pandemic scales up.

**Figure 5:**
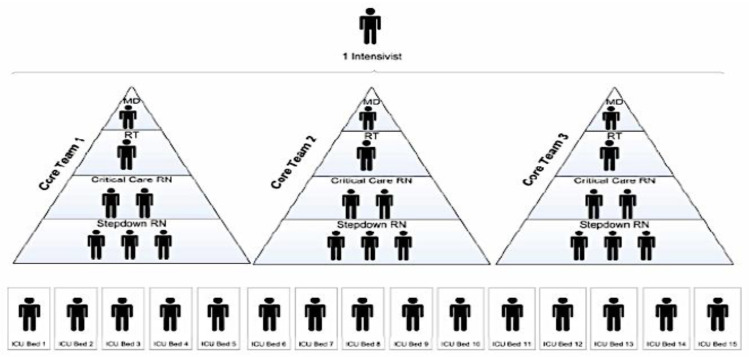
Model of ICU care teams.

**Figure 6:**
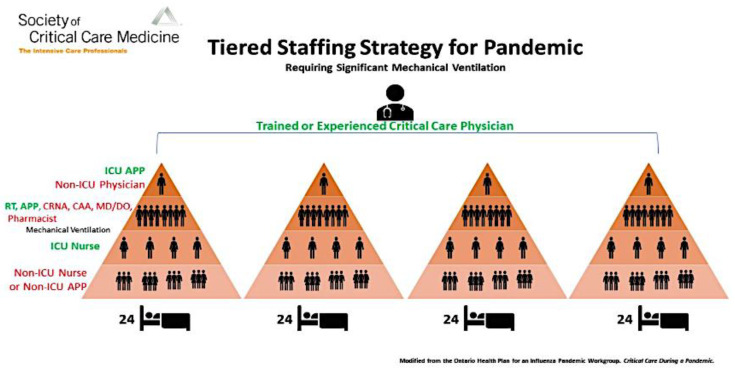
Expanded application of tiered staffing strategy for pandemic.

A Team-Based Approach Outlines Care Being Provided by Teams of Healthcare Professionals for Groups of Patients (Figure 4, Figure 5 and Figure 6). 

The team is led by an ICU physician who works with a respiratory therapist trained in critical care and 2 ICU nurses who supervised 3 step-down nurses. Each team provides care for 15 patients [12]. 

In this model, one experienced ICU physician oversees 4 teams composed of ICU physicians, respiratory therapist, and nurses supported by other hospital professionals to take care of 24 patients each [10].

#### 3.3.1. Rationale

To overcome the anticipated shortage of ICU staff during the COVID-19 pandemic, hospitals are recommended to adopt a team-based approach. In the Ontario Health Plan for an Influenza Pandemic Care Team Approach, and the Society of Critical Care Medicine (SCCM) Tiered Staffing Strategy for a Pandemic are recommended models for ICU staff augmentation strategies during pandemics such as COVID-19. Both strategies have similar concepts and applications. They focus on the utilization of non-experienced healthcare workers to work in collaboration (in teams) with experienced staff to increase the capacity of care for critically ill patients. This strategy demonstrated to work effectively in pandemic situations [7,21]. 

The tiered staffing strategy combines experienced ICU nurses with reassigned hospital nurses. Instead of the regular care delivery model where each ICU nurse provides care for one to two patients (Figure 2), in this strategy, each ICU-trained nurse will supervise and direct other two re-assigned nurses who have useful skills but lack experience in the ICU setting to ultimately provide care for four critically ill patients. ICU physician(s) trained in critical care or those who regularly manage ICU patients will oversee all nurse teams (Figure 3) [12,13,21,22]. 

As the situation unfolds, teams can be expanded to care for more patients such as six or eight or more as required. Tiered staffing models are not set standards and each hospital must determine the best combination of staff based on their resources [11,23,24]. Combining experienced and non-experienced ICU-trained nurses will help to ensure adequate levels of care and not overwhelm ICU-trained staff. When implementing the current strategy and combining inexperienced team members, it is recommended to maintain effective communication among the team. This can be achieved through utilizing different ways such as team huddles at the start of each shift and at regular intervals, such as every 4 hours, to discuss team assignments, patient care goals, and red flags that should be reported immediately to the team leader [25]. This will ensure effective communication and allows each team member to discuss his/her patients’ needs and get the experts’ opinion. If a physical huddle is difficult, virtual huddles can be applied to enhance patients’ safety and to keep all team members aware of all updates and changes in the unit [25]. 

#### 3.3.2. Applications of a Team-Based Approach

The report of the Ontario Health Plan for an Influenza Pandemic presented an example of a tiered strategy and called it Care Team Model (Figure 4). In this model, healthcare workers who have useful skills but lack experience in critical care can work in teams supervised by experienced staff and collectively care for a larger group of patients. In place of an individual specialized nurse caring for one to two patients, a team of mixed experienced nurses provides the care for a group of patients. This is possible because in combination, they have the complete skills set and pertinent experience required to care for expanded patient numbers. In this example, one intensivist can supervise three teams, each composed of one physician, one respiratory therapist and two ICU nurses who supervised three step-down nurses. Each one of the 3 teams will take care of 5 patients and the 3 teams together will provide care to 15 patients [10,11]. The care team model focuses on the provision of care by a team of healthcare workers. Teams would be created with feedback loops and operate under this designated hierarchy and guided by expected job functions and responsibilities. This model has proven to be effective in past emergencies [10,11,15,16].

The SCCM presented an expanded example of the applications of tiered staffing strategy for pandemics with a larger number of healthcare workers and larger capacity for care provision (Figure 5). It suggests that one ICU-experienced physician oversees the care of 4 teams, and each team provides care for 24 patients. Each one of these teams is supervised by an ICU physician or non-ICU physician such as an anesthesiologist, pulmonologist, surgeon, or hospitalist, who does not frequently perform ICU care but has some ICU training. Each team is composed of an experienced respiratory therapist and other clinicians such as physicians, nurse anesthetists, or pharmacists who are experienced in managing ventilated patients. There are four ICU nurses in each team; each nurse is responsible for supervising the other three re-assigned nurses and each re-assigned nurse will care for two patients. Ultimately each team will provide care for 24 patients and the four teams together will provide care for 96 patients [16]. This strategy is an alternative strategy that may be implemented as ICU-trained nurses fall ill and ICU-trained nurses become less available to care for patients.

### 3.4. Recommendation 4: Training Model for ICU Tiered Staffing Strategy for COVID-19 Pandemic

**Figure 7:**
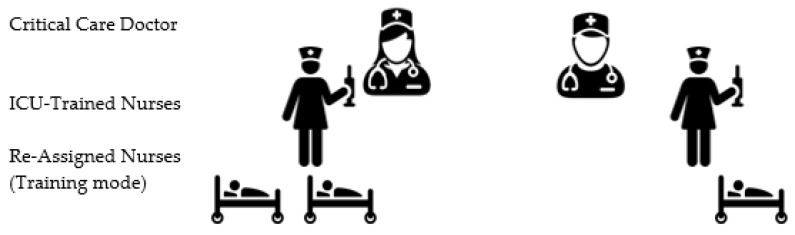
Training preparation for tiered staffing strategy for COVID-19 pandemic preparation.

Illustrated in this model (Figure 7) is a team composed of two ICU nurses; each nurse trains one re-assigned nurse and together they provide care for two critically ill patients. Training should only be added for the re-assigned nurse to care for two patients (hopefully, at least one of which is ventilated) under the direction of an ICU-trained nurse. This will orient the re-assigned nurse as well as orient the ICU-trained nurse as to what tasks and responsibilities will be assigned, divided, and shared. In the training, ventilator management should be the main focus, including modalities, high PEEP considerations, O2 saturations, ABG interpretation, suctioning, proning, sedation, paralytics, and pain control, though sedation vacations must be reviewed by medical staff as to risk versus benefit. 

#### Rationale 

A significant number of critically ill patients will be admitted to intensive care units during the COVID-19 pandemic. Staffing will be further strained by the threat of experienced ICU staff nurses becoming ill [26]. During the COVID-19 pandemic, it is anticipated that the projected shortfall of well-trained ICU nurses will impact the care of critically ill ventilated patients. Consequently, the focus should not be only to increase the numbers of mechanical ventilators but must also address the number of trained critical care nurses required to care for mechanically ventilated COVID-19 patients, alongside non-COVID-19 patients requiring ICU care [25,26]. Assigning hospital nurses to work immediately in ICU during crisis time without enough training may put the nurse and patients at high risk. Therefore, planning for appropriate nursing staff prior to such a pandemic is required. Augmenting critical care nursing staff is one innovative way to scale up staffing capacity during a pandemic. Individual healthcare organizations must modify their strategies thereby aligning ICU staffing with their patient needs and with available resources [25,26]. In this strategy, consideration should be made to have already chosen and delegated non-ICU-trained nurses to be stationed in the ICU and be assigned to an ICU nurse in order to form a controlled baseline training prior to the actual surge. This will establish roles and responsibilities and form the foundation to build an expanding team when a surge becomes evident. 

## 4. Conclusions

In anticipation of COVID-19 demands upon nursing staff and subsequent potential weakening of care levels in the provision of patient care, specifically in the ICU setting, a panel was formed to raise and answer critical concerns. The nursing surge capacity of critically ill patients with COVID-19 in the ICU was addressed through searching available evidence. Substantiation was retrieved from a variety of databases inclusive of published and unpublished studies. The retrieved studies were then reviewed by a minimum of two reviewers independently using JBI critical appraisal tools. The recommendations in the recent guidelines covered ICU nursing surge capacity strategies. We recommend that hospitals implement the evidence-based strategies that have been shown to be effective such as a team-based approach, and to establish other innovative strategies for ICU nursing staff surge capacity in the COVID-19 pandemic. As new evidence presents itself, further updates of the guideline will be issued.

## References

[1] Alhazzani W., Al-Suwaidan F., Al Aseri Z., Al Mutair A., Alghamdi G., Rabaan A., Algamdi M., Alohali A., Asiri A., Alshahrani M. (2020). The saudi critical care society clinical practice guidelines on the management of COVID-19 patients in the intensive care unit. Saudi Crit. Care J..

[2] Wu Z., McGoogan J.M. (2020). Characteristics of and important lessons from the coronavirus disease 2019 (COVID-19) outbreak in China: Summary of a report of 72 314 cases from the Chinese Center for Disease Control and Prevention. JAMA.

[3] Livingston E., Bucher K. (2020). Coronavirus disease 2019 (COVID-19) in Italy. JAMA.

[4] Milton D.K., Fabian M.P., Cowling B.J., Grantham M.L., McDevitt J.J. (2013). Influenza virus aerosols in human exhaled breath: Particle size, culturability, and effect of surgical masks. PLoS Pathog..

[5] World Health Organization Corona Virus Disease (COVID-19) Dashboard. https://covid19.who.int.

[6] World Health Organization Rational Use of Personal Protective Equipment for Coronavirus Disease 2019 (COVID-19) Interim Guidance 27 February 2020. https://apps.who.int/iris/bitstream/handle/10665/331215/WHO-2019-nCov-IPCPPE_use-2020.1-eng.pdf.

[7] World Health Organization Coronavirus. https://www.who.int/health-topics/coronavirus#tab=tab_1.

[8] Chu D.K., Akl E.A., Duda S., Solo K., Yaacoub S., Schünemann H.J., Chu D.K., Akl E.A., El-harakeh A., Bognanni A. (2020). Physical distancing, face masks, and eye protection to prevent person-to-person transmission of SARS-CoV-2 and COVID-19: A systematic review and meta-analysis. Lancet.

[9] World Health Organization Advice on the Use of Masks in the Context of COVID-19. https://www.who.int/publications/i/item/advice-on-the-use-of-masks-in-the-community-during-home-care-and-in-healthcare-settings-in-the-context-of-the-novel-coronavirus-(2019-ncov)-outbreak.

[10] Society of Critical Care Medicine United States Resource Availability for COVID-19. https://sccm.org/getattachment/Blog/March-2020/United-States-Resource-Availability-for-COVID-19/United-States-Resource-Availability-for-COVID-19.pdf?lang=en-US.

[11] Aragon Penoyer D. (2010). Nurse staffing and patient outcomes in critical care: A concise review. Crit. Care Med..

[12] Ontario Health Plan for an Influenza Pandemic Care Critical Care During a Pandemic. http://www.cidrap.umn.edu/sites/default/files/public/php/21/21_report.pdf.

[13] Maves R.C., Downar J., Dichter J.R., Hick J.L., Devereaux A., Geiling J.A., Kissoon N., Hupert N., Niven A.S., King M.A. (2020). Triage of scarce critical care resources in COVID-19—An implementation guide for regional allocation: An expert panel report of the task force for mass critical care and the American College of Chest Physicians. Chest.

[14] Chung W., Sohn M. (2018). The impact of nurse staffing on in-hospital mortality of stroke patients in Korea. J. Cardiovasc. Nurs..

[15] The University of Toronto Interdepartmental Division of Critical Care Medicine Working Group Management Principles of Adult Critically Ill COVID-19 Patients. https://criticalcare.utoronto.ca/file/180/download?token=E8_KA4WU.

[16] Murthy S., Gomersall C.D., Fowler R.A. (2020). Care for critically Ill patients with COVID-19. JAMA.

[17] Ajao A., Nystrom S.V., Koonin L.M., Patel A., Howell D.R., Baccam P., Lant T., Malatino E., Chamberlin M., Meltzer M.I. (2015). Assessing the capacity of the US health care system to use additional mechanical ventilators during a large-scale public health emergency. Disaster Med. Pub. Health Prep..

[18] Kleinpell R.M., Grabenkort W.R., Kapu A.N., Constantine R., Sicoutris C. (2019). Nurse practitioners and physician assistants in acute and critical care: A concise review of the literature and data 2008–2018. Crit. Care Med..

[19] McHugh M.D., Ma C. (2013). Hospital nursing and 30-day readmissions among medicare patients with heart failure, acute myocardial infarction, and pneumonia. Med. Care.

[20] Cho E., Chin D.L., Kim S., Hong O. (2016). The relationships of nurse staffing level and work environment with patient adverse events. J. Nurs. Scholarsh..

[21] American College of Chest Physicians Surge Priority Planning COVID-19: Critical Care Staffing and Nursing Considerations. http://www.chestnet.org/Guidelines-and-Resources/Resources/Surge-Priority-Planning-COVID-19-Critical-Care-Staffing-and-Nursing-Considerations.

[22] Scott D., Irfan U., Kirby J. The Next Coronavirus Crisis Will Be A Shortage of Doctors and Nurses. https://www.vox.com/2020/3/26/21192191/coronavirus-us-new-york-hospitals-doctors-nurses.

[23] Society of Critical Care Medicine Critical Care Statistics. https://www.sccm.org/Communications/Critical-Care-Statistics.

[24] Driscoll A., Grant M.J., Carroll D., Dalton S., Deaton C., Jones I., Lehwaldt D., McKee G., Munyombwe T., Astin F. (2017). The effect of nurse-to-patient ratios on nurse-sensitive patient outcomes in acute specialist units: A systematic review and meta-analysis. Eur. J. Cardiovasc. Nurs..

[25] Kleinpell R.M. (2014). ICU workforce: Revisiting nurse staffing. Crit. Care Med..

[26] North Carolina Healthcare Foundation Strategies to Support Nursing Surge Capacity during Biological Event. https://www.ncbon.com/vdownloads/coronavirus/nursing-surge-capacity-resource.pdf.

